# Ultrahigh Performance C_60_ Nanorod Large Area Flexible Photoconductor Devices via Ultralow Organic and Inorganic Photodoping

**DOI:** 10.1038/srep05041

**Published:** 2014-05-23

**Authors:** Rinku Saran, Vlad Stolojan, Richard J. Curry

**Affiliations:** 1 Advanced Technology Institute, Department of Electronic Engineering, University of Surrey, Guildford, Surrey, GU2 7XH, United Kingdom

## Abstract

One dimensional single-crystal nanorods of C_60_ possess unique optoelectronic properties including high electron mobility, high photosensitivity and an excellent electron accepting nature. In addition, their rapid large scale synthesis at room temperature makes these organic semiconducting nanorods highly attractive for advanced optoelectronic device applications. Here, we report low-cost large-area flexible photoconductor devices fabricated using C_60_ nanorods. We demonstrate that the photosensitivity of the C_60_ nanorods can be enhanced ~400-fold via an ultralow photodoping mechanism. The photodoped devices offer broadband UV-vis-NIR spectral tuneability, exhibit a detectivitiy >10^9^ Jones, an external quantum efficiency of ~100%, a linear dynamic range of 80 dB, a rise time 60 µs and the ability to measure ac signals up to ~250 kHz. These figures of merit combined are among the highest reported for one dimensional organic and inorganic large-area planar photoconductors and are competitive with commercially available inorganic photoconductors and photoconductive cells. With the additional processing benefits providing compatibility with large-area flexible platforms, these devices represent significant advances and make C_60_ nanorods a promising candidate for advanced photodetector technologies.

One dimensional (1D) nanostructures have received significant interest in recent years for their potential application in a variety of flexible optoelectronic devices including field effect transistors^1^, light emitting diodes^2^, logic gates^3^, photovoltaic cells^4^, sensors^5^ and photodetectors^6^. In particular for photodetector applications, intensive research is currently being focused on 1D nanostructures. However, most of the photodetectors reported are fabricated using 1D inorganic nanostructures that have several limitations which restricts their use in practical device applications^7^. These include requiring a high temperature growth process, offering a limited range of spectral sensitivity, poor device stability and incompatibility with large scale device fabrication and integration. In contrast, reports of 1D organic nanostructures for photodetection are rarer with the majority of these describing single nanowire devices^8^,^9^,^10^,^11^. To date there is no report of 1D organic nanostructures that offer rapid room temperature synthesis, are compatible with large-area and flexible substrate device fabrication, can be fabricated and operated in air, and present figures of merit that can compete with inorganic materials and with commercially available photodetectors. Amongst candidate organic materials, single-crystal organic semiconductors are of particular importance as they exhibit superior optoelectronic properties, such as long range exciton diffusion^12^, can offer air stability^13^,^14^, excellent charge carrier mobility^15^ and high photoconductivity^16^. The potential applications of photosensitive 1D organic nanostructures is broad encompassing electrophotography^17^, spectroscopy^18^, optical switches^19^, colorimetery^20^, X-ray detection^21^ and organic optocouplers^22^.

Here, we report large-area flexible photoconductor devices fabricated using 1D C_60_ nanorods. The devices display photosensitivity across the UV-vis-NIR spectrum with a detectivity, D*, of >10^9^ Jones. To achieve this remarkable level of performance we introduced an ultralow photodoping mechanism, using both organic and inorganic photodopants, which significantly enhances the photosensitivity of the C_60_ nanorods. Upon excitation these photodopants fulfill two roles. Firstly they fill trap states in the C_60_ nanorods via interfacial electron transfer thereby increasing the photoconductivity. Secondly, once all trap states are filled, the transferred electrons significantly contribute to the overall photocurrent generation while the holes remain trapped in the photodopant. These two processes enable devices to achieve an external quantum efficiency *EQE(λ)* ~100%. We find that photodoping increases the detectivity of C_60_ nanorod devices by up to 2 orders of magnitude to reach a detectivity >10^9^ Jones, whilst also displaying a wide linear dynamic range (LDR) of 80 dB along with photodetection capability up to modulation frequencies of ~250 kHz. These figures of merit obtained for C_60_ nanorod devices makes them highly attractive for a low-cost photodetector technology. In addition, we demonstrate that these photodetectors can be fabricated on flexible substrates using a simple planar interdigitated electrode architecture under ambient air conditions at room temperature. Such planar photodetectors can be easily integrated with complementary metal-oxide-semiconductor (CMOS) technology, and unlike photodiodes they only require a single metal electrode deposition step. They also eliminate the need to use transparent substrates or electrodes thus reducing the overall cost of the photodetector.

## Results

**Figure 1:**
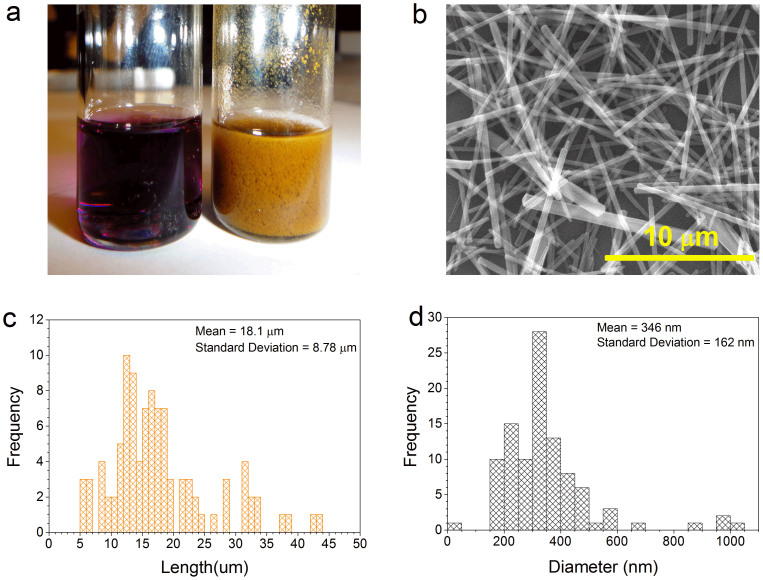
C_60_ nanorods preparation. (a) Photograph of C_60_ dissolved in m-xylene (left) and C_60_ nanorods (right) prepared via the fast liquid-liquid interfacial precipitation (FLLIP) method. The optical absorption of both C_60_ in m-xylene and C_60_ nanorods is shown in figure 2d. (b) Scanning electron microscope (SEM) image of 1D C_60_ nanorods obtained via the FLLIP method. (c) Histogram of C_60_ nanorod length distribution with a mean length of 18 µm. (d) Histogram of C_60_ nanorod diameter distribution with a mean diameter of 350 nm.

**Figure 2:**
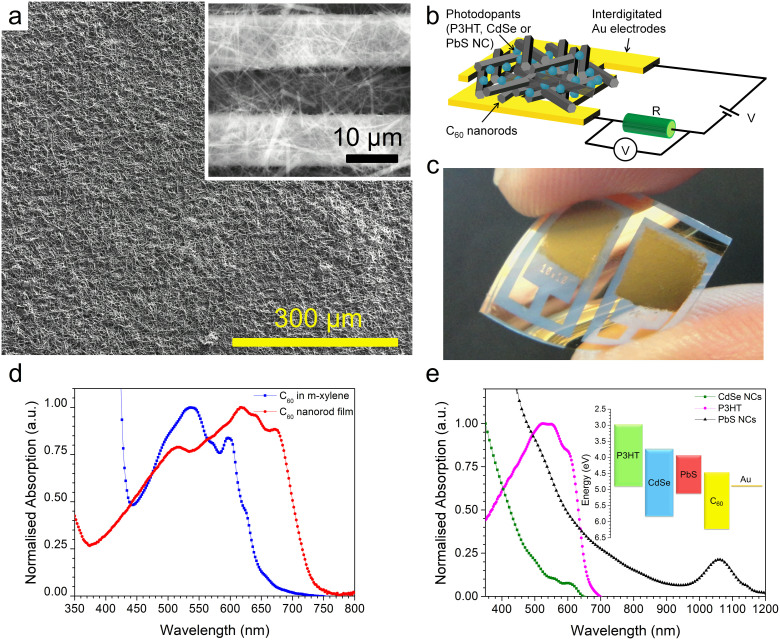
C_60_ nanorod photoconductor device architecture and absorption. (a) A 5 kV SEM image of a typical C_60_ nanorod photoconductor fabricated via depositing a C_60_ nanorod film onto pre-patterned interdigitated Au electrodes. Inset to (a) is a 30 kV SEM image showing C_60_ nanorods bridging 10 µm wide interdigitated electrodes with an inter-electrode spacing of 10 µm. (b) A schematic depicting the planar device architecture employed for fabricating photoconductor devices and the electrical circuit diagram used to characterize the devices. (c) Photograph of two typical C_60_ nanorod photoconductor devices fabricated on a flexible PEN substrate. The active area covers the region of interdigitated electrodes which measures 5 × 5 mm. (d) Normalised optical absorption spectra of C_60_ dissolved in m-xylene and a C_60_ nanorod film. C_60_ nanorods display broadened and extended absorption, red shifted by ~100 nm, compared to C_60_ in solution. (e) Normalised optical absorption spectra of P3HT, CdSe NCs, and PbS NCs used for photodoping the C_60_ nanorod devices. Inset to (e) is a schematic of the relative energy level alignment between C_60_ and the photodopants. The relative energy level alignment strongly favours photoexcited electron transfer from the photodopants to the C_60_ with holes remaining trapped in the photodopant.

**Figure 3:**
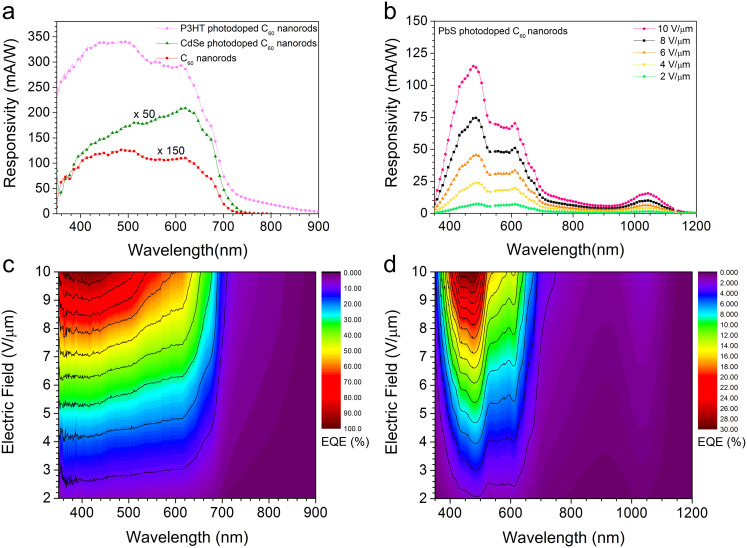
Spectral responsivity and external quantum efficiency of C_60_ nanorod photoconductor devices. (a) Relative comparison of spectral responsivity obtained for C_60_ nanorod only and P3HT or CdSe NC photodoped C_60_ nanorod devices. Responsivity was measured using an applied electric field of 10 Vµm^-1^, and a modulation frequency of 235 Hz. All devices display photosensitivity across the full visible spectrum with the photodoping significantly enhancing the responsivity by up to 2 orders of magnitude. (b) Electric field dependent spectral responsivity of PbS NC photodoped device. The responsivity spectrum follows the C_60_ absorption spectrum in the visible region, confirming that the ultralow photodoping provided via PbS NCs enhances the photosensitivity of C_60_ nanorods. The spectral responsivity is extended by the use of PbS NC to provide UV-vis-NIR sensitivity. (c) The EQE of a P3HT photodoped device as a function of applied electric field and wavelength displaying a peak EQE approaching ~100% at 415 nm. (d) The EQE of a PbS NC photodoped device as a function of applied electric field and wavelength. It can be seen that EQE of the device is strongly field dependent in the region where the C_60_ nanorods absorb (350–750 nm) whilst in spectral range (750–1150 nm) the EQE remains below ~2%, even with increase in the applied electric field.

C_60_ nanorods were prepared via the fast liquid-liquid interfacial precipitation (FLLIP) method^23^. C_60_ powder (purity 99.9%) was dissolved in m-xylene to form a solution with concentration ~2.2 mg mL^−1^. The C_60_ solution (2 ml) was then slowly added to 3 mL of isopropyl alcohol (IPA) with the solution instantly turning a brownish-yellow color indicating the formation of a suspension of 1D C_60_ nanorods. Figure 1a shows a photograph of C_60_ dissolved in m-xylene and the C_60_ nanorods obtained via FLLIP method. FLLIP method facilitates rapid large scale preparation of C_60_ nanorods at room temperature and takes less than 10 seconds. A SEM image of the 1D C_60_ nanorods obtained using the FLLIP method is shown in figure 1b. The mean length and diameter of C_60_ nanorods was found to be 18 µm and 350 nm respectively, figure 1 c,d. Planar photoconductor devices were fabricated via depositing a C_60_ nanorod film onto 10 µm wide interdigitated gold (Au) electrodes with 10 µm spacing, on flexible polyethylene-2,6-naphthalate (PEN) substrates. A SEM image of a typical as deposited C_60_ nanorod film is shown in figure 2a with a schematic illustration of the device architecture employed in figure 2b. A photograph of a set of two typical C_60_ nanorod flexible photoconductor devices is shown in figure 2c. The optical absorption spectra of a C_60_ dissolved in m-xylene and of a C_60_ nanorod film are shown in figure 2d. The C_60_ nanorods display a significantly broadened and extended absorption spectrum in comparison with C_60_ to provide spectral coverage up to 750 nm. The large (~100 nm) redshift of the absorption in the solid state with respect to solution is attributed to enhanced Coulombic interaction between the C_60_ molecules^24^. The photoconductor devices fabricated were characterized to evaluate the performance of the C_60_ nanorods as a photosensitive material. The responsivity, *R*(*λ*), of a photoconductor is defined as the ratio of the photocurrent generated, *I_ph_*, to the incident optical power, *P_light_*, equation (1): 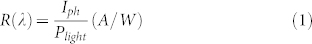
The spectral responsivity of C_60_ nanorod photoconductor devices at an applied electric field of 10 Vµm^−1^ is shown in figure 3a. Photosensitivity from 350 to ~730 nm is observed, closely following the C_60_ nanorod absorption spectrum, with a maximum responsivity of 0.85 mAW^−1^ obtained ~488 nm. The external quantum efficiency (*EQE*(*λ*)) can be obtained from *R*(*λ*)using the equation (2): 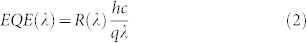
Where, *h* is Plank's constant, *c* is the speed of light, *q* the elementary charge and *λ* the wavelength of incident light. To compare photodetectors of varying active area and device geometry the detectivity, D*, figure of merit is often used and is given by equation (3): 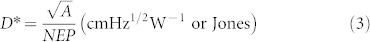
Where, *A* is the active area of the device and *NEP* is the noise equivalent power defined as the optical power at which the signal-to-noise ratio (SNR) is unity, and thereby giving the limit of detection, and is obtained using equation (4): 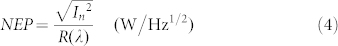
Here, *I_n_* is the spectral noise current density and *R*(*λ*) is the responsivity. The C_60_ nanorod photoconductor device exhibits a peak D* of ~3.8 × 10^7^ Jones at 488 nm. This is surprisingly high detectivity for 1D organic material considering that the devices were fully fabricated and characterized in air without any optimization. Typically, due to the tendency of C_60_ to randomly crystallize when used in solution, C_60_ film deposition is usually carried out by vacuum evaporation restricting its application in many devices and adding to fabrication cost. In contrast, C_60_ nanorods offer compatibility with solution processing, including roll-to-roll processing, and contact printing. The high photosensitivity extending across the full visible spectrum clearly demonstrates the significant potential of C_60_ nanorods in other applications as well including photovoltaic cells and electrophotography.

In highly ordered molecular crystals there is a large orbital overlap between adjacent molecules which can lead to efficient charge transport and high charge carrier mobilities^16^. However, when molecular crystal based devices operate in air they suffer severe degradation due to the formation of trap states (Supplementary Information Fig. S4). C_60_ is a n-type semiconductor and the introduction of traps in C_60_ due to intercalation of oxygen is well-known^24^,^25^. Thus, when the C_60_ nanorod photoconductor device is irradiated with photons the majority of excited electrons are localized in the trap states rather than contributing to the photocurrent, reducing the responsivity. This can be addressed by the injection of additional charge carriers to fill these trap states either by increasing the applied bias or through illumination of the devices with a high light intensity such that the number of excited electrons exceeds the density of traps (Supplementary Information Fig. S6). These approaches, however, limit the dynamic range of the device. An ideal alternative to circumvent these problems is to use a photodopant in combination with such trap rich molecular crystals. A photodopant must form a type-II heterojunction (for electron photodoping) such that when the composite is photoexcited the photodopants provide the additional electrons to fill C_60_ traps states, via interfacial electron transfer, and importantly may also contribute to overall photocurrent. A further benefit of photodoping is that it can significantly improve the photo-oxidation stability, assist in determining trap state distribution and can extend the spectral sensitivity of the device^26^,^27^,^28^.

To investigate the effect of photodoping on C_60_ nanorod photosensitivity, we used variety of materials including the organic π-conjugated polymer Poly(3-hexylthiophene-2,5-diyl) (P3HT), rhodamine 6G (R6G), 7,7,8,8-tetracyanoquinodimethane (TCNQ) and inorganic semiconductor NCs (CdSe and PbS). The optical absorption spectrum of the photodopants is shown figure 2e and supplementary information figure S1, along with the relative energy level alignment to C_60_. Photodoping was achieved via sensitizing C_60_ nanorods with ultralow volumes of photodopants. The responsivity spectra obtained for P3HT and CdSe NC photodoped photoconductor devices is shown in figure 3a. A significant (~50-fold) increase in responsivity is found upon photodoping with CdSe NCs yielding a maximum responsivity of 42 mAW^−1^ at ~617 nm. The use of P3HT as the photodopant provides a ~400-fold improvement in the responsivity peaking at ~340 mAW^−1^. The spectral responsivity of the PbS NCs photodoped devices as a function of applied electric field and wavelength is shown in figure 3b. These devices also display a significant improvement in responsivity with a maximum value of 115 mAW^−1^ at ~480 nm, whilst also displaying broadband UV-vis-NIR photosensitivity from 350 nm to 1150 nm.

## Discussion

It should be noted that the photodoped devices we report here work on a fundamentally different mechanism as compared to more commonly reported planar heterojunction donor/acceptor (D/A) devices reported previously^29^,^30^,^31^,^32^,^33^. In typical blend devices the relative donor to acceptor volume ratio is very high and the devices rely primarily on the absorption and conductivity of the donor material. Here, the photodopant donor materials are utilized differently such that they enhance the photosensitivity of the acceptor material i.e. the C_60_ nanorods. This is achieved via an ultralow photodoping mechanism. A transmission electron microscope (TEM) image of ultralow photodoped C_60_ rod with CdSe NCs is shown in supplementary information figure S5. Conventional molecular n-type doping of C_60_ films has been shown to fill C_60_ traps and thereby increase the electron mobility by more than 3 orders of magnitude^34^. In extending this concept to active photodoping we demonstrate that the photosensitivity of the 1D C_60_ nanorods can be enhanced significantly such that devices approach responsivity values close to commercial photodetectors. Moreover, such a type of ultralow photodoping enables devices to achieve a photoconductive gain.

To validate that the enhancement observed in the responsivity of the doped devices occurs due to a photodoping mechanism, we compare the responsivity spectrum of all the photodoped devices with that of the undoped C_60_ nanorods device, Figure 3a,b. It is evident that the responsivity of the photodoped devices is increased by up to two orders of magnitude as compared to the undoped C_60_ nanorod device. Interestingly, the responsivity spectrum of all the three photodoped devices still display the similar peaks at ~520 nm, ~620 nm and ~680 nm, which were observed in the undoped C_60_ nanorods. These peaks correspond to the characteristic absorption peaks of C_60_ and are also present in the optical absorption of the C_60_ nanorod film, Fig 2e^24^. This strongly suggests that C_60_ nanorods remain the primary origin of the photocurrent even in photodoped devices. The role of the additional electrons supplied by the photodopants is to pacify the deep trap states present in the C_60_ nanorods, thereby increasing the C_60_ electron mobility^26^,^35^. These results along with previous reports^34^,^35^ confirm that in C_60_ films and C_60_ nanorods electron transport is hampered due to presence of high density of traps. However, via an ultralow doping mechanism and careful selection of dopants and photodopants, both mobility and the photoconductivity of such trap rich molecular organic semiconductors can be enhanced by orders of magnitude. It is observed that in the CdSe NC photodoped device there is an additional enhancement in the responsivity between ~550 nm and 650 nm corresponding to the CdSe NC absorption. This implies that CdSe NCs are donating sufficient electrons to saturate the C_60_ trap states with a surplus of donated electrons directly contributing to the photocurrent.

**Figure 4:**
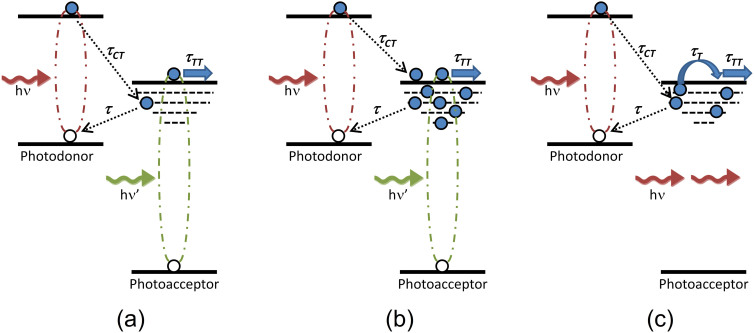
Schematic illustration of photodoping mechanism in donor-acceptor photoconductor devices. (a) Device operation under low-intensity irradiation in which both the donor (e.g. P3HT/CdSe NCs/PbS NCs) and acceptor (e.g. C_60_ nanorods) are able to absorb the incident light. Electron transfer, with a rate 1/τ_CT_, from the donor occurs contributing to the filling of acceptor trap states and increasing the electron mobility of the acceptor. The trapped electron may then recombine with the hole in the donor (with rate 1/τ) returning the electron mobility to its original value. This process leads to an increased charge carrier concentration and facilitates the photoexcited electrons generated in the acceptor to contribute to photocurrent, which in the absence of photodopant get localized in the traps. (b) As the traps are filled, electrons transferred from the donor are also able to contribute to the photocurrent and the electron mobility in the acceptor is further enhanced. (c) Irradiation at wavelengths below the acceptor bandgap results in trap filling only occurring via electron transfer from the photodonor. Transferred electrons may contribute to the photocurrent when trap saturation occurs or *via* thermalisation (with rate 1/τ_T_) from trap states prior to this. In (a) and (b) recombination of trapped electrons with the hole in the acceptor is also possible.

We now discuss the working mechanism of the organic and inorganic photodoped devices, using P3HT as an example. When the photoconductor device is photoexcited, excitons are generated in both P3HT and C_60_, Figure 4a. The relative energy level alignment between P3HT and C_60_ forms a type-II heterojunction and strongly favors photoinduced electron transfer from P3HT to C_60_. The transfer of holes from the photodopant (P3HT or the semiconductor NCs) to C_60_ is energetically forbidden, figure 2e. In commonly reported planar photoconductors fabricated using blends the donor material may provide a conducting pathway for holes to reach the electrodes and thereby preventing photoconductive gain being achieved. In our photodoped devices hole transport is blocked and the photocurrent generation is solely due to the flow of electrons, extracted by applying an electric field. Upon photoexcitation, the electrons from the photodopant are transferred to C_60_ nanorods via the interfacial electron transfer process. As discussed above these transferred electrons primarily are utilized to fill the high density of traps present in the C_60_ nanorods. The transferred electrons may recombine back with the ground state parent hole via the interfacial recombination process. As the C_60_ traps begin to fill, subsequently the Fermi energy level is also raised towards the C_60_ lowest unoccupied molecular orbital (LUMO). Meanwhile, this process facilitates the photoexcited electrons generated in C_60_ to fully contribute to photocurrent, which were previously localized in the absence of any photodopant, and this is clearly reflected in the enhanced responsivity spectra of all the doped devices. Once all the traps are filled, that is when the number of donated electrons equals the density of traps, the Fermi level approaches the bottom of the C_60_ LUMO. At this point any subsequently donated electrons from the photodopants contribute to the overall photocurrent generation, Figure 4b. Thus, a remarkable enhancement in the responsivity of photodoped devices is observed. Inspection of the spectral responsivity of P3HT doped devices shows photosensitivity between 750 nm and 900 nm despite neither material absorbing light beyond 750 nm. This NIR extension of the spectral responsivity is consistent for all in P3HT photodoped devices and its origin was found to be electric field dependent. A similar voltage dependent NIR response has been seen before in P3HT based photodetectors^36^ and is consistent with the presence and charge transfer filling/excitation of deep localized trap states^37^. It should be emphasized that the responsivity values obtained for the P3HT photodoped C_60_ nanorod device are higher than commercially available silicon carbide (SiC) or gallium nitride (GaN) ~200 mAW^−1^ and close to silicon (Si) ~500 mAW^−1^ photodetectors used for UV and visible light detection respectively with the added advantage of large-area, flexible substrate compatibility and room temperature processing.

In order to gain deeper insight of the photodoping mechanism in C_60_ nanorods a photodopant was chosen such that it absorbs light beyond the optical absorption range of C_60_ nanorods. PbS NCs display a size-tunable optical absorption from near UV to NIR via quantum confinement and are widely used in photovoltaic cells and photodetectors to harness NIR photons^38^,^39^. The spectral responsivity of the PbS NCs photodoped device is shown in figure 3b as a function of applied electric field. It can be seen that in the spectral range where both PbS NCs and C_60_ nanorods absorb (350–750 nm) the device displays a high responsivity, closely following the C_60_ nanorod absorption spectrum. In this spectral range the working mechanism of the device is similar to that of P3HT or CdSe NCs doped device (Figure 4a,b), and further confirms that the ultralow photodoping provided by PbS NCs also enhances the photoconductivity of C_60_ nanorods. However, in the spectral range 750–1150 nm C_60_ nanorods do not absorb photons and hence electron-hole pairs are only generated in PbS NCs, Figure 4c. Thus when exciting in this spectral region the C_60_ nanorods regain their intrinsic nature of high density of traps. Typical trap densities reported for C_60_ films are in the range of 10^18^–10^19^ cm^−3^ 34,40. However the trap density in 1D C_60_ nanorods may be expected to exceed this due to their large surface-to-volume ratio. Following photoexcitation of PbS NCs only in the 750–1150 nm spectral range, electrons from PbS NCs are transferred to C_60_. The majority of the transferred electrons are now utilized in filling C_60_ nanorod traps, thus the photocurrent is reduced and, the responsivity of the device falls sharply beyond ~750 nm. Nonetheless, a moderate photocurrent remains peaking at ~1060 nm corresponding to the first exciton absorption peak of PbS NCs. This suggests that it is possible to saturate the C_60_ nanorod trap states via photodoping. Alternatively, thermalisation of trapped carriers into the C_60_ LUMO may also be possible thus providing a contribution to the photocurrent. It is also observed that the spectral responsivity of the PbS NC doped device only displays a strong electric field dependence in the spectral region in which the C_60_ nanorods absorb (350–750 nm). This confirms that charge transport in these photodoped photoconductor devices occurs through C_60_ nanorods and they are the origin of the electric field dependent photocurrent in these devices. The study on other materials used as photodopants including R6G and TCNQ is provided in the supplementary information.

The *EQE(λ)* of the P3HT and PbS NCs photodoped device is shown in figure 3 c,d. Up to an applied electric field of 3 Vµm^−1^, the *EQE(λ)* remains below ~10% and ~2% for P3HT and PbS NCs photodoped devices, respectively. However, for both devices the *EQE(λ)* is significantly enhanced across the visible region as the applied electric field is increased, reaching maximum values of ~100% at 415 nm and ~30% at 471 nm for the P3HT and PbS NC photodoped devices at 10 Vµm^−1^. It is noticeable that in the spectral range (730–1150 nm) the *EQE(λ)* of the PbS NCs photodoped device remains below ~2% even with the increase in the applied electric field. This suggests that for C_60_ nanorod photoconductors to operate efficiently, photoexcited electrons from both photodopant and the C_60_ nanorods must contribute to the overall photocurrent generation. Nevertheless, unlike conventional organic photodiodes, which cannot achieve an *EQE(λ)* ~100%, unless phenomenon such as singlet exciton fission is exploited^41^, P3HT photodoped photoconductor approaches *EQE(λ)* ~100% and is among the best reported for organic single-crystal large area devices^16^. Typically in organic heterojunction photodiodes the absorption of a photon leads to the creation of a Coulombically bound exciton. The photoexcited electron may overcome this Coulomb attraction provided that the energy level offset between the donor and the acceptor is sufficient. The electron is then transferred to the acceptor whilst the hole dissociates towards the opposite electric contact. Thus for a single absorbed photon the electron and hole are collected at opposite electrodes thereby restricting the internal quantum efficiency of such devices below 100%. Contrary to photodiodes, the planar photoconductors we report here can exhibit a photoconductive gain due to trapping of one of the charge carriers^42^. The gain of a photoconductor is defined as the ratio of the exciton lifetime to the charge carrier transit time given by the equation (5): 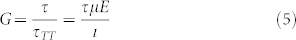
where, *G* is the photoconductive gain*, τ* is the lifetime of trapped charge carrier, *τ_TT_* is the transit time, *μ* is the mobility, *ι* is the distance between electrodes and *E* is the applied electric field. From equation (5) it is clear that gain can be realized either by increasing the lifetime of the trapped carriers or by reducing the transit time of the mobile carriers (e.g. via increasing the applied electric field). The passivation of C_60_ nanorod trap states by the use of photodopants enhances the electron mobility in C_60_ nanorods thus enabling the high responsivities obtained and the possibility of achieving gain. Using an average value for the electron mobility in C_60_ single-crystals^15^ of ~5 cm^2^V^−1^s^−1^ the transit time, *τ_TT_*, can be estimated as ~2 ns for applied electric field strength of 10 Vµm^−1^. As the holes are trapped in the photodopant the mobile electrons can circulate many times in the circuit providing τ > τ_TT_ which may be achieved under the influence of an applied electric field. It should be noted that although the *EQE(λ)* of the device approaches ~100% at high applied fields, it typically remains below ~60% across the whole spectral range up to an applied electric field of 8 Vµm^−1^. A sharp rise in *EQE(λ)* between 350–540 nm is observed when the field is increased to 10 Vµm^−1^. This increase in *EQE(λ)* in this region is a distinctive signature behavior of electric field-dependent photoconductivity in C_60_^43^,^44^. Previous studies of planar C_60_ devices (operating as a photoconductor due to hole trapping) reported a dramatic increase in *EQE(λ)* in this region from ~30% at 4.5 Vµm^−1^ to ~5000% at 35 Vµm^−1^ and was attributed to photomultiplication phenomena^44^,^45^. It is also widely reported that photoexcited carrier generation in C_60_ occurs due to Frenkel exciton (intramolecular) disassociation when exciting above ~530 nm, whilst when exciting in the spectral range 350–540 nm the origin of photoexcited carriers is due to electric field assisted dissociation of charge transfer (CT) states (intermolecular)^46^. It is within this region that we observe the increase in *EQE(λ)* to 100% in our devices and it might therefore be associated with this mechanism.

**Figure 5:**
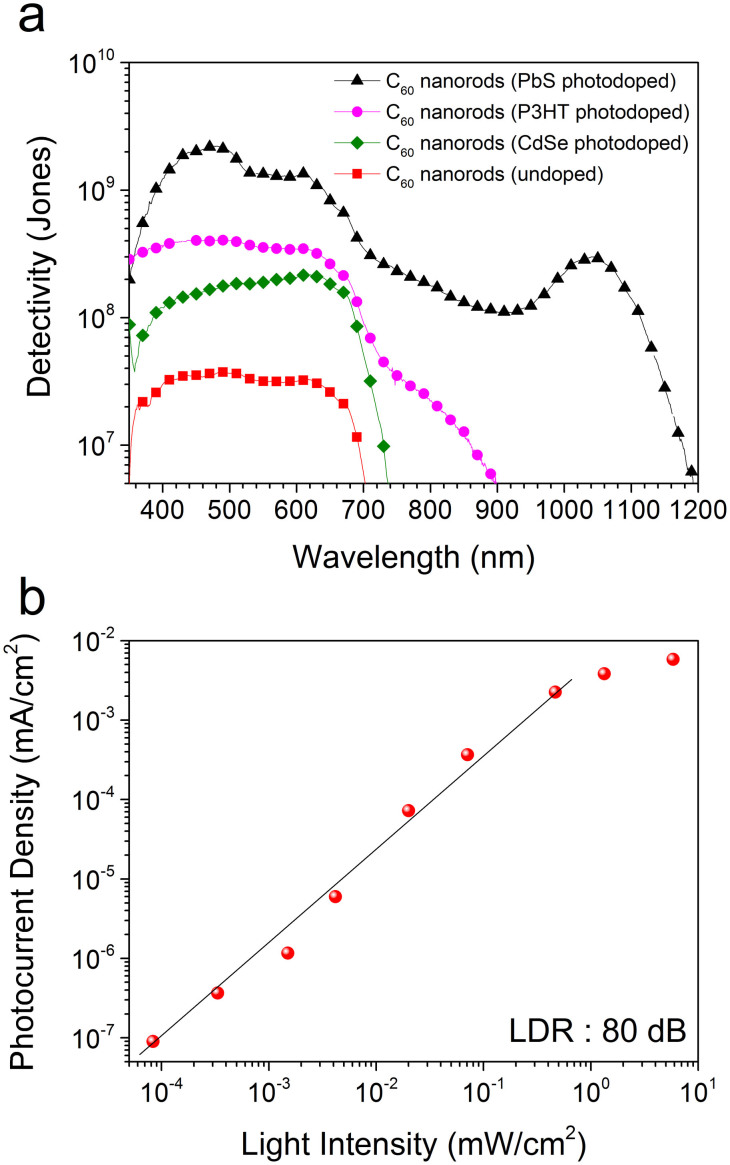
Detectivity and linear dynamic range of C_60_ nanorod photoconductor devices. (a) Relative comparison of detectivities of C_60_ nanorod only and photodoped photoconductor devices as a function of wavelength. P3HT and CdSe NC sensitized devices exhibit a detectivity of ~10^8^ Jones representing an order of magnitude increase over C_60_ nanorod only photoconductor devices. PbS NC photodoped devices exhibit the highest detectivity of ~10^9^ Jones. (b) The photocurrent as a function of incident optical power measured using modulated (235 Hz) 514 nm incident light. The device shows a dynamic range covering 5 orders of magnitude (100 dB), within which linearity is maintained for 4 orders magnitude, corresponding to a linear dynamic range of ~80 dB.

The detectivity of the C_60_ nanorod only and the photodoped C_60_ nanorod photoconductor devices is shown in figure 5a obtained at an electric field of 10 Vµm^−1^, modulation frequency of 235 Hz, and for an active area of 25 mm^2^. The photodoped photoconductor devices show greater than an order of magnitude increase in detectivity over the undoped devices. An increase in peak detectivity from 3.8 × 10^7^ Jones to 2.2 × 10^8^, 4 × 10^8^, and 2.3 × 10^9^ Jones is obtained when doping with CdSe NCs, P3HT and PbS NCs respectively, and are for example suitable for low cost imaging and video applications^47^,^48^. The linear dynamic range (LDR) of a photoconductor is defined as the range over which the photocurrent increases linearly with the incident optical power and is expressed in decibels (dB). The dynamic range of the photodoped C_60_ nanorod device is found to extend over five orders of magnitude (100 dB) within which a LDR of 80 dB (over four orders of magnitude) is found. This 80 dB LDR is above that required for most imaging applications and is even higher than reported inorganic and hybrid photoconductor device^39^,^49^. Typically, the photoconductive gain in reported inorganic materials rely on electron trapping and mobile holes, the inverse to how our device operates. As a result when these inorganic material-based devices are excited with high light intensities, a reduction in photocurrent gain due to filling of traps occurs. This leads to a fall in responsivity which in turn restricts the device LDR to low light intensities. In our devices photocurrent generation is due to flow of electrons and the gain is dependent on the C_60_ excitonic lifetime and the photodopant hole trapping. As a result excellent device responsivity is maintained even under high light intensities and thereby providing a wide LDR. The observed LDR exceeds the requirements for the replacement of charge-coupled device (CCD) imaging sensors, is above that of commercially available GaN (50 dB)^50^ and InGaAs (66 dB)^51^ photodetectors, and very close to that of Si (120 dB) photodetectors^51^.

**Figure 6:**
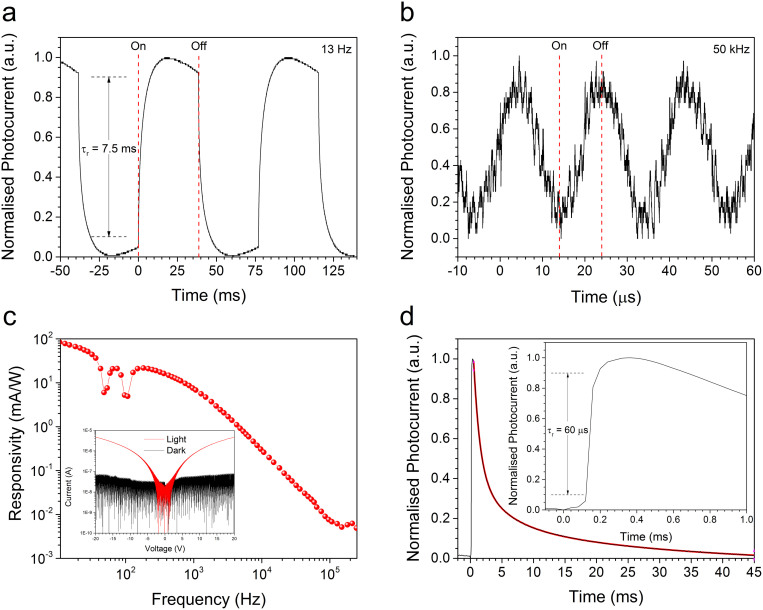
Temporal response of C_60_ nanorod photoconductors. (a) The transient photocurrent response of a typical photodoped C_60_ nanorod photoconductor device measured under ~514 nm illumination (~312 µWcm^−2^) modulated at 13 Hz with an applied electric field of 10 Vµm^−1^. The device exhibits a rise time of 7.5 ms. (b) The photocurrent response of the same device under the identical measurement conditions except for an increase in the modulation frequency to 50 kHz. (c) Spectral responsivity of the photoconductor device as a function of modulation frequency measured at an applied electric field of 3 Vµm^−1^. The photoconductor shows a typical decrease in the responsivity with increase in frequency. The features at ~50 Hz and ~100 Hz correspond to notch filters applied by the lock-in amplifier to remove line-frequency artefacts. The device displays the ability to measure ac signals up to ~250 kHz (the limit of the lock-in amplifier). Inset to (c) is the current-voltage characteristic of the device under dark and light conditions. (d) The transient response of photoconductor device measured under 510 nm pulsed (~8 ns at a 21 Hz repetition rate) illumination. The device displays a fast rise time of 60 µs (inset) and a slow decay indicating the presence of long lived trap states in the optically active material composite. The solid red line represents a fit consisting of the sum of three exponential decays.

In many applications, such as optical switching, the photoconductor temporal response is of high importance. The normalized photocurrent response of a typical photodoped device under modulated ~514 nm excitation at 13 Hz and 50 kHz is shown in Figure 6a,b. The rise time of the photoconductor, defined as the time required for the photocurrent to rise from 10% to 90% of its final value, was measured to be 7.5 ms which again is well suited to many applications. The temporal response of the device is a function of incident optical intensity, applied electric field, measurement bandwidth and the dopant concentration. The dependence of spectral responsivity of the device on modulation frequency is shown in Figure 6c. The device displays a typical reduction in responsivity with increasing frequency. The responsivity of the device falls from ~85 mA/W at 10 Hz to ~1 mA/W at 5 kHz, though the device demonstrates signal detection up to modulation frequencies as high as 250 kHz (limited by the lock-in amplifier bandwidth) which is in the regime required for high-frequency photoconductive switches. The current-voltage characteristic of the devices is shown inset to figure 6c. In photodoped devices interfacial recombination process may be significant enabling devices to achieve high responsivity and high speed operation, simultaneously^52^. To further quantify, the transient response a PbS NC photodoped photoconductor device was measured using a 510 nm, 8 ns pulsed laser source (operating at 21 Hz), at an applied field of 3 Vµm^−1^ figure 6d. The device shows a fast rise time of 60 µs and a slow decay indicating the presence of long lived trap states in the device. The decay transient shows tri-exponential decay with decay constants of 0.99 ± 0.01 ms, 4.4 ± 0.1 ms, and 27.4 ± 0.1 ms suggesting that there are three different relaxation processes occurring. The fast temporal response together with broad spectral coverage makes C_60_ nanorod photoconductors promising candidates for high dynamic range (HDR) large-area flexible image sensing technology.

To summarize, we have shown that low-cost large-area flexible photoconductors can be fabricated employing C_60_ nanorods using a room temperature solution processable and rapid procedure undertaken in air. C_60_ nanorods intrinsically are highly photosensitive, however the introduction of high density of traps primarily due to intercalation of oxygen drastically affect their photosensitivity. This was addressed by introducing an ultralow organic and inorganic photodoping mechanism which not only enhances the photosensitivity of C_60_ nanorods by orders of magnitude but also enables devices to achieve photoconductive gain. The figures of merit achieved for C_60_ nanorods are among the highest reported for 1D organic nanostructures and even exceed commercially available photodetectors. These C_60_ nanorod photoconductor devices represent a significant advance in organic photoconductor technology and their compatibility with large-area flexible device architectures makes them highly attractive for variety of photodetection applications.

## Methods

### Photoconductor Device Fabrication

Au interdigitated electrodes, 10 μm wide and with inter-electrode spacing of 10 µm (total device area = 5 mm × 5 mm) were patterned on a flexible polyethylene-2,6-naphthalate (PEN) substrate via ultraviolet (UV) lithography. Prior to use the substrates were cleaned using acetone and isopropyl alcohol (IPA) respectively for 10 minutes using ultrasonication. Primer and photoresist (Rohm and Hass MICROPOSIT S1813) were then subsequently spin coated onto the substrates at 3500 rpm for 30 seconds. After depositing the photoresist, the substrates were baked on a hot plate (80°C for 1 min). The photoresist coated on the substrates was then exposed to UV light through a pre-patterned mask at an energy dose of ~120 mJ cm^−2^. The pattern was developed by immersing the UV exposed substrates in the developer (Rohm and Hass MF319 developer) for 3 minutes. Following this, the substrates were washed using de-ionized water, dried with nitrogen, and placed within a sputterer for metal deposition. An adhesion layer (~6 nm) of Titanium (Ti) was sputtered first before deposition of ~120 nm of gold (Au). After metal deposition the substrates were immersed in acetone for the lift-off process to produce the desired interdigitated pattern. C_60_ nanorod films were then deposited onto the interdigitated planar electrodes from the C_60_ nanorods solution. Photodoping was achieved via sensitizing C_60_ nanorods with ultralow volumes (~250 nL) of photodopants P3HT (~6 mg mL^−1^ in toluene), R6G (~0.5 mg mL^−1^) CdSe NCs (~2.2 mg mL^−1^ in toluene), or PbS NCs (~8 mg mL^−1^ in toluene).

### Photoconductor Device Characterization

To obtain the device responsivity, *R(λ),* photocurrent from the biased devices was extracted by measuring the voltage drop across a load resistor connected in series with the device using lock in amplification. Illumination to the devices was provided using chopped monochromatic light obtained from 100 W tungsten lamp and monochromator. Optical filters were used to prevent second order grating reflections illuminating the devices. The optical power falling on the device was measured independently using calibrated Si and InGaAs detectors. Dynamic range measurements were performed using the same set up, illumination to the photoconductor device was given using an Argon ion laser operating at ~514 nm and with the light intensity falling onto the device controlled by varying the laser power. To measure noise current, *I_n_*, the devices were placed in an electrically shielded dark box, biased, and the total noise current was measured using a lock in amplifier. The reference frequency was provided by a signal generator with the lock-in amplifier acting as a narrow bandwidth amplifier directly outputting the total noise current. The noise equivalent power *(NEP)* of the devices was calculated by dividing the measured noise current by the responsivity under identical measurement conditions that is, for the same modulation frequency and the applied electric field. The detectivity, D*, of the devices were calculated by dividing the square root of the active area of the device by the *NEP*. The detectivity of the photoconductor devices reported was obtained for an applied electric field of 10 Vµm^−1^ at a modulation frequency of 235 Hz and for an active area of 25 mm^2^. Frequency dependent transient measurements were recorded using 514 nm Argon ion laser irradiation modulated using an acousto-optic modulator (AOM). The modulation frequency was controlled using a square-wave function generator. The transient response of the devices to pulsed 510 nm illumination was undertaken using a ~8 ns pulse from a Spectra-Physics VersaScan OPO operating at 21 Hz. Transient response measurements were performed with the device biased and the transient photocurrent signal recorded by measuring the voltage drop across a resistor using a digital oscilloscope. Current-voltage characteristics of the devices were measured using a precision source measure unit. Absorption spectra were obtained using a Varian Cary 5000 UV-Vis-NIR spectrophotometer.

All methods including C_60_ nanorods preparation, device fabrication and device characterization were performed in air at room temperatures.

## Author Contributions

R.S. prepared the devices. V.S. performed (HR)TEM studies and analysis. R.S. and R.J.C. performed the device characterization. R.S., V.S. and R.J.C. prepared the manuscript and figures.

## Supplementary Material

Supplementary Information Supplementary Information
