# Supplementary material for: Ultrahigh Performance C60 Nanorod Large Area Flexible Photoconductor Devices via Ultralow Organic and Inorganic Photodoping
Source: Sci Rep. 2014 May 23;4:5041. doi: 10.1038/srep05041 (PMC4031472; doi:10.1038/srep05041)
Supplement: Supplementary Information [file srep05041-s1.pdf]

Supplementary Information

Ultrahigh Performance C_{60} Nanorod Large Area Flexible Photoconductor Devices via Ultralow Organic and Inorganic Photodoping

*Rinku Saran and Richard J Curry**

Advanced Technology Institute, Department of Electronic Engineering, University of Surrey, Guildford, Surrey, GU2 7XH, United Kingdom. E-mail: r.j.curry@surrey.ac.uk

Photodoping C_{60} nanorods with Rhodamine 6G (R6G) and 7,7,8,8-tetracyanoquinodimethane (TCNQ).

In order to gain deeper insight into the device operation we have studied the effect of additional photodopants on C_{60} nanorod device performance by employing rhodamine 6G (R6G) and 7,7,8,8-tetracyanoquinodimethane (TCNQ) as dopant materials.

R6G is a well-known n-type organic dye and has been previously employed as a photosensitizer to enhance the photosensitivity of devices.[1-3] The absorption spectra of R6G in methanol is shown in figure S1a along with its relative energy level alignment with C_{60} in figure S1b. C_{60} nanorod devices sensitized with R6G (0.5mg/ml in methanol) were also found to increase the responsivity of the device. A comparison of spectral responsivity of the C_{60} and R6G photodoped C_{60} device is shown in figure S2b. The photocurrent is seen to be enhanced particularly in the spectral region above ~575 nm, where C_{60} only absorbs, demonstrating the benefits of the photodoping provided by R6G filling C_{60} traps (Figure S2a). A contribution to the photocurrent from R6G can also be seen in the 500 nm to 550 nm with a shoulder in the responsivity spectrum at ~520 nm corresponding to the peak of the R6G absorption.

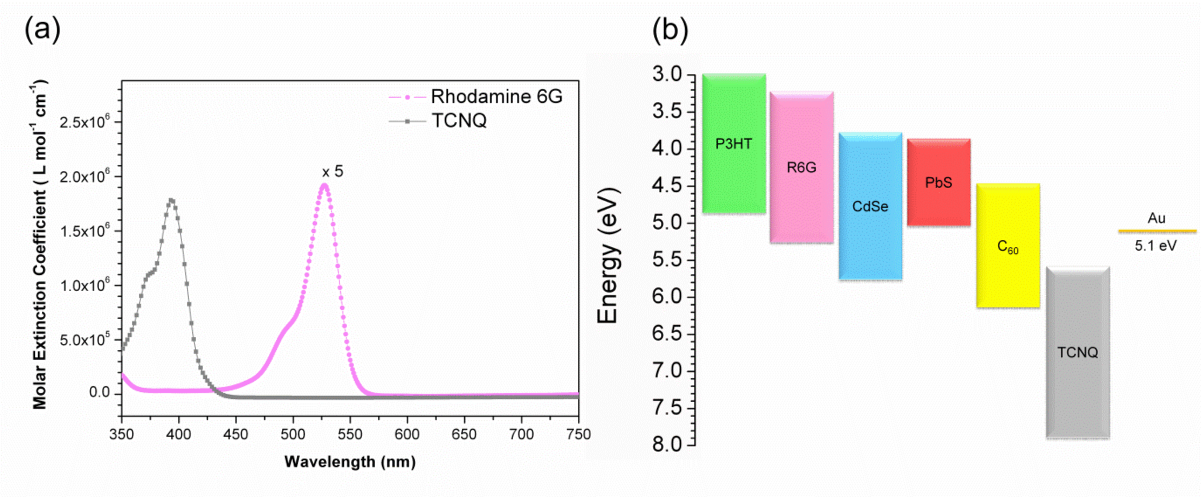

Figure S1. Absorption and energy level alignment (a) Absorption spectrum of R6G in methanol and TCNQ in acetonitrile (b) Relative energy level alignment between C_{60} and variety of materials used in the study to photodope C_{60} nanorods. Unlike other dopant materials used, TCNQ doesn't form a type-II heterojunction with C_{60}, thus photoinduced electron transfer from TCNQ to C_{60} is energetically forbidden. The energy level values for R6G and TCNQ are taken from reference [2] and [4] respectively.

The transient response of C_{60} rod only devices and R6G photodoped devices obtained using pulsed (~8 ns pulse width at 21 Hz repetition rate) 520 nm illumination are shown in S2d. It can be seen that the photocurrent obtained from the C_{60} rods only device decays faster as compared to R6G photodoped devices indicating a direct dependence of the photocurrent generation process on the photodopant. This, along with the enhanced spectral response, precludes the increase in photocurrent arising solely from the dopant reducing contact resistance between C_{60} rods for example.

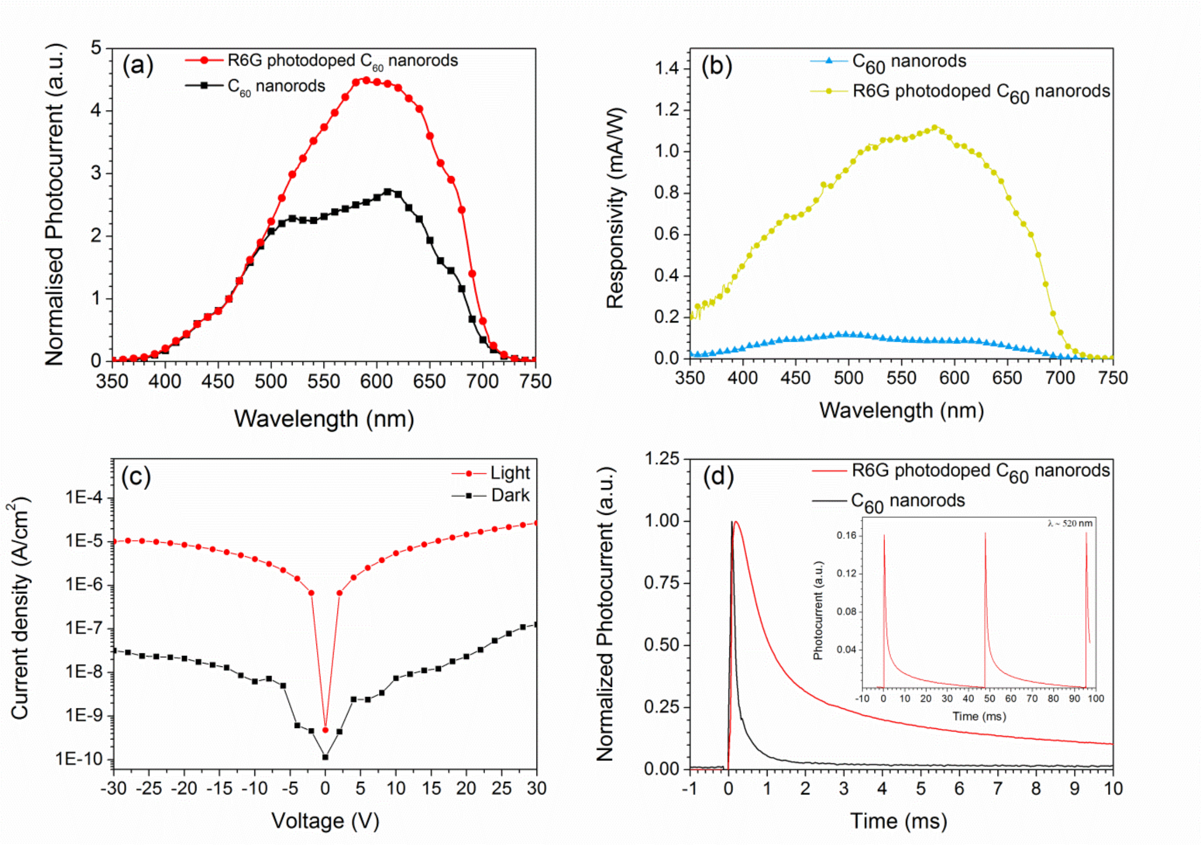

Figure S2. Photodoping C_{60} nanorods with rhodamine 6G (a) Normalised photocurrent (at 460 nm) of C_{60} nanorods only and R6G photodoped devices. (b) Spectral responsivity of C_{60} nanorod and R6G photodoped C_{60} nanorod devices measured at an applied electric field strength of 1V/μm. (c) J-V characteristic of the R6G photodoped C_{60} nanorod device. (d) Normalized transient photocurrent response of C_{60} nanorod device before and after photodoping with R6G. Photocurrent in the R6G photodoped decays slowly relative C_{60} rods indicating the dopant hole trapping process. Inset to (d) is shown the full transient response of the R6G photodoped device. Transient decays were obtained using a ~8 ns pulse at 21 Hz repetition rate exciting at ~520 nm (1.2 mW) with the device biased with a 1V/μm electric field.

Conversely when TCNQ was used as the 'photodopant' a reduction in device photocurrent was observed across the entire spectral region (Figure S3a). Inspection of the relative energy level

alignment between C_{60} and TCNQ shows that the TCNQ lowest unoccupied molecular orbital (LUMO) lies just above the mid-point of the C_{60} LUMO and highest occupied molecular orbital (HOMO). As such excitation of TCNQ will not then allow electron transfer into C_{60} trap states located below its LUMO and therefore we would not expect any increase in photocurrent as observed for the other photodopants utilised. Furthermore, it can be seen that in addition to TCNQ absorbing photons, which therefore will not contribute to the photocurrent, it is possible for excited electrons within C_{60} to relax into the unexcited TCNQ LUMO. These effects result in the reduction of the photocurrent as observed and further support the proposed mechanism behind the operation of the devices reported within. We note that devices formed from TCNQ alone show a weak photoresponse matching the TCNQ absorption spectrum (Figure S3b).

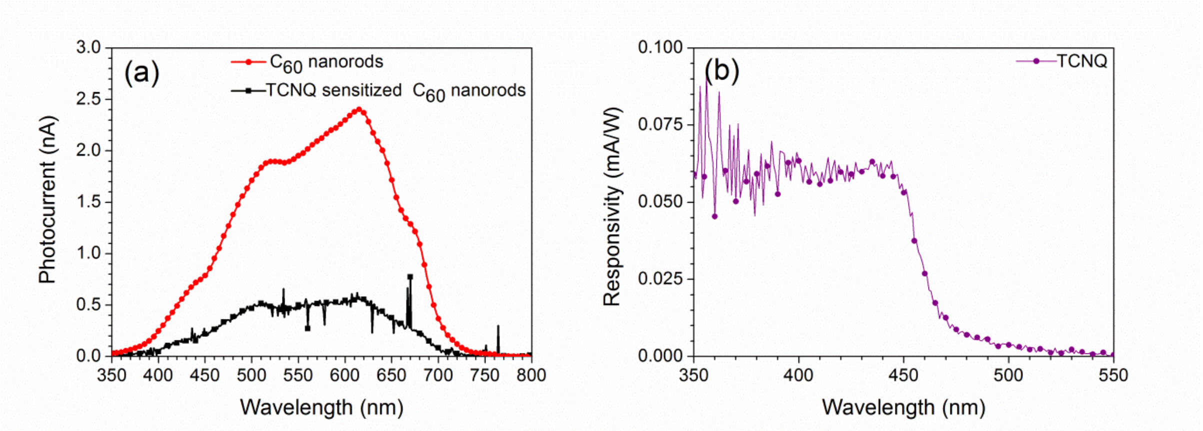

Figure S3. Photodoping C_{60} nanorods with TCNQ. (a) Photocurrent quenching observed in C_{60} nanorod device upon 'photodoping' with TCNQ. (b) Spectral responsivity of a TCNQ only device measured at an electric field of 10 V/μm.

Effect of oxygen on C_{60} device performance

C_{60} crystals intrinsically show good conductivity however this is significantly reduced upon exposure to oxygen. For example, C_{60} crystals prepared via solvent evaporation and C_{60} nanorods prepared via LLIP method have shown very high electron mobilities with reported values of 11 $cm^2V^{-1}S^{-1}$ and 1 $cm^2V^{-1}S^{-1}$ respectively, when measured in nitrogen environment.[5,6] To demonstrate this, we fabricated a C_{60} nanorod device in nitrogen and performed I-V measurements under nitrogen for ~ 30 minutes before exposing the same device to air (Figure S4). The device exhibits an initially high conductivity in nitrogen which over ~30 minutes does

not degrade significantly. Upon exposure to air a significant decrease in conductivity is observed followed by a further decrease until after ~2.5 hours the current stabilized. We note that all of the devices reported in this study were fabricated in air under room light and therefore it is likely that this effect is the origin of the traps which photodoping process fills.

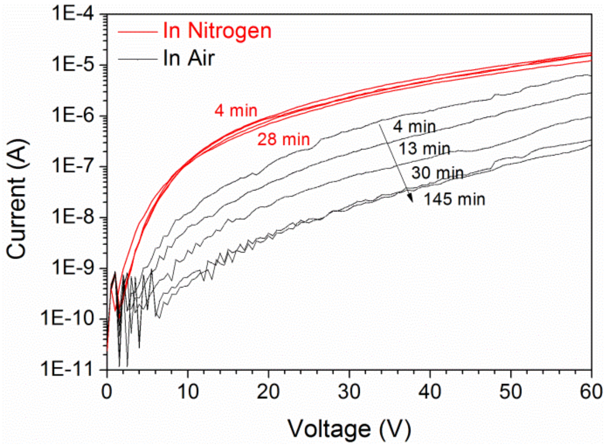

Figure S4. Effect of air exposure on conductivity of C_{60} nanorod device. Dark I-V characteristics of a typical C_{60} nanorod device fabricated and operated in nitrogen prior to exposure to air.

HRTEM Studies of Photodoped C_{60} Rods

In Figure S5 we show bright and dark field TEM images of CdSe NCs photodoped C_{60} rods. The bright field image shows the CdSe NCs dispersed on the surface of the faceted C_{60} crystal; at the edge of the crystal (top) we observe an apparent monolayer coverage of the NCs, as we look down a facet of the C_{60} crystal. The Z-contrast image confirms that the small dark structures are not pores, but have a higher effective Z. The bright field image also shows lattice fringes parallel to the axis of the C_{60} crystal at (1.05±0.05) nm, corresponding to the 110 family of planes (ref: ICSD 602518). The inset shows a high resolution image of a CdSe nanocrystal, with lattice fringes at (3.14±0.05)Å, which identifies it as the 111 planes in the cubic CdSe, space group Fm-3m (ref: ICSD 181026). Characterisation of the C_{60} rods (Figure S5 right) revealed their single crystal nature similar to that previously reported.[7]

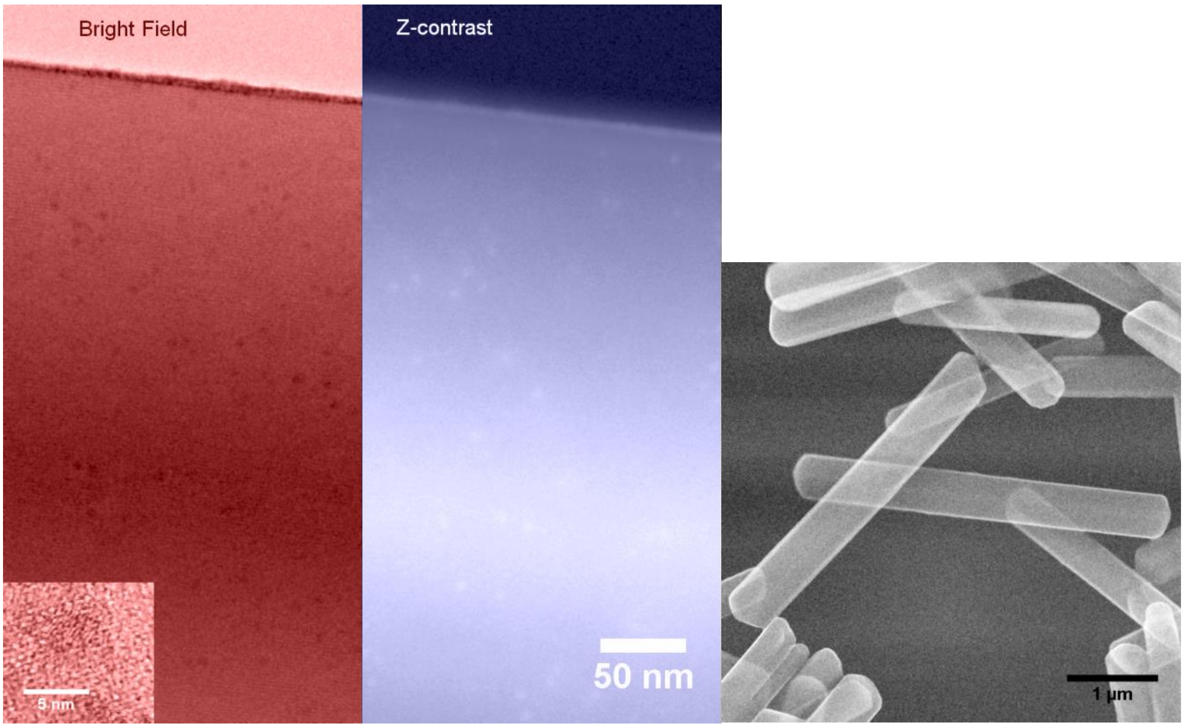

Figure S5. TEM image of a C_{60} crystal decorated with CdSe NCs. (left) A composite image obtained using various imaging modes in a Hitachi HD2300A Field-Emission Scanning Transmission Electron Microscope. (right) Secondary electron image of C_{60} rods.

Methods for enhancing photocurrent

Our results clearly show that the conductivity of C_{60} nanorods decreases drastically with exposure to oxygen, which is due to the creation of trap states that hamper the hopping transport mechanism within the C_{60} nanorods (figure S4). We further demonstrate that the hopping process can be made efficient in C_{60} rods by filling electron traps states. As discussed in the manuscript this can be done by increasing the charge carrier concentration in C_{60} in three different ways: (i) by increasing the applied bias; (ii) by increasing the optical intensity; and (iii) by using photodopants (including PbS and CdSe nanocrystals, P3HT and rhodamine 6G).

In Figure S6 we demonstrate these methods using PbS NCs as the photodopant material. In Figure S6a increasing the applied electric field is seen to lead to a direct increase in photocurrent. Similarly, increasing the incident light intensity at a fixed bias also increases the photocurrent (Figure S6b). Finally, figure S6c shows directly the effect of increasing the PbS NC concentration on increasing the photocurrent. The increase in the dopant concentration (PbS NCs) also increases the spectral sensitivity in the near-IR region. It is evident that this increase in responsivity of the device is due to increased electron transfer from the PbS NCs to C_{60}. The

peak in the near-IR region (~ 880nm) in the spectral responsivity corresponds to the first excitonic absorption of the nanocrystals used. Figure S6d shows the I-V characteristics of the higher PbS NC doping concentration device in the dark and under light (620nm) conditions.

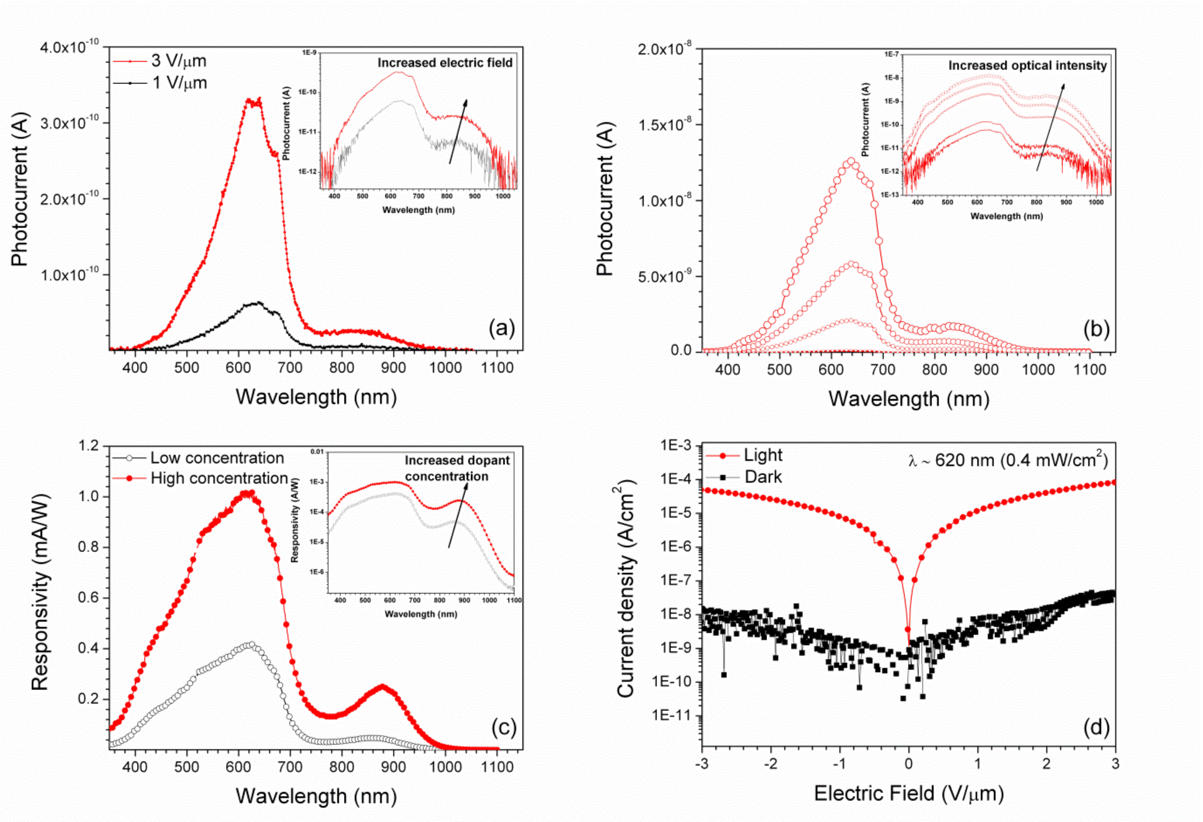

Figure S6. Effect of varying electric field, optical intensity and photodopant concentration. (a) Photocurrent obtained from a typical PbS NC photodoped C_{60} rod device using applied fields of 1 and 3 V/μm. (b) Increase in the spectral photocurrent obtained via increasing the optical power intensity. (c) Spectral responsivity measured at an electric field of 1V/μm (235 Hz) of the C_{60} photodoped device at low and high photodopant (PbS NCs) concentrations. It can be seen that by increasing the dopant concentration responsivity of the device can be enhanced both in the visible and in the near-IR region (d) J-V characteristic of the high PbS NC doping concentration device under dark and light (620nm) conditions.

For completeness we also provide typical J-V characteristics of all photodoped devices obtained under dark conditions. It can be observed that upon doping the dark current is increased typically depending upon the type of donor material. We note that as device fabrication and photodoping process was undertaken by simple dropcasting procedures, dark currents in devices were found to be varying using the same photodopant materials.

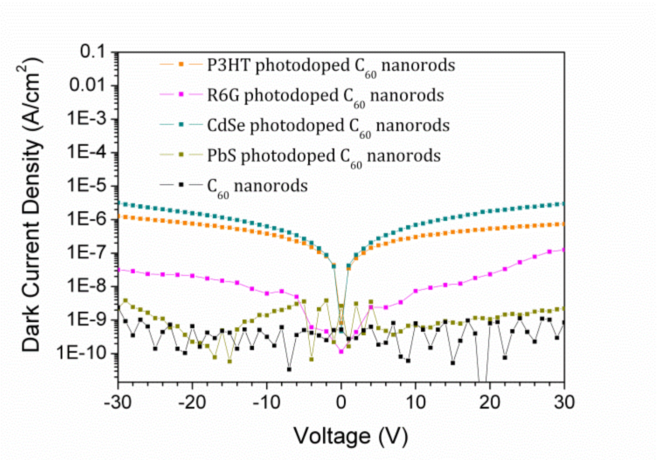

Figure S7. Dark current density. Dark current densities of a typical C_{60} nanorod and photodoped devices.

References

1 Somani, P. & Radhakrishnan, S. Sensitization effect in conducting polyaniline by rhodamine 6G. *Journal of Materials Science: Materials in Electronics* **13**, 735-741, (2002).

2 Fukuda, T., Kimura, S., Honda, Z. & Kamata, N. Solution-Processed Green-Sensitive Organic Photoconductive Device Using Rhodamine 6G. *Molecular Crystals and Liquid Crystals* **566**, 67-74, (2012).

3 Singh, H. K. *et al.* Study of swift heavy ion irradiation effect on Rhodamine 6G dye for dye sensitize solar cell application. *Vacuum* **87**, 21-25, (2013).

4 Nordin, M. N., Bourdakos, K. N. & Curry, R. J. Charge transfer in hybrid organic-inorganic PbS nanocrystal systems. *Physical Chemistry Chemical Physics* **12**, 7371-7377, (2010).

5 Li, H. *et al.* High-Mobility Field-Effect Transistors from Large-Area Solution-Grown Aligned C_{60} Single Crystals. *Journal of the American Chemical Society* **134**, 2760-2765, (2012).

6 Barzegar, H. R., Larsen, C., Edman, L. & Wågberg, T. Solution-Based Phototransformation of C_{60} Nanorods: Towards Improved Electronic Devices. *Particle & Particle Systems Characterization* **30**, 715-720, (2013).

7 Jin, Y. *et al.* Structural and optoelectronic properties of C_{60} rods obtained via a rapid synthesis route. *Journal of Materials Chemistry* **16**, 3715-3720, (2006).
